# Effects of Ocean Acidification and Temperature Coupling on Photosynthetic Activity and Physiological Properties of *Ulva fasciata* and *Sargassum horneri*

**DOI:** 10.3390/biology13080640

**Published:** 2024-08-21

**Authors:** Kai Wang, Xiang Tao, Shouyu Zhang, Xu Zhao

**Affiliations:** 1 College of Oceanography and Ecological Science, Shanghai Ocean University, Shanghai 201306, China; kwang@shou.edu.cn (K.W.); 18957451910@163.com (X.T.); syzhang@shou.edu.cn (S.Z.); 2 Research Center of Marine Ranching, Shanghai Ocean University, Shanghai 201306, China

**Keywords:** macroalgae, ocean acidification, greenhouse effect, chlorophyll fluorescence

## Abstract

**Simple Summary:**

Macroalgae in natural marine areas play an important role in mitigating ocean climate change. The complexity of natural conditions also makes it necessary to study macroalgae not only by considering the effects of changes in a single factor but also by exploring the coupled effects of different environmental conditions on macroalgae. Therefore, in this study, two species of macroalgae were used as experimental subjects to observe their growth processes under different co-treatments of temperature and CO_2_ concentration. The results of this study can provide a reference for how natural macroalgae can cope with future changes in ocean climate.

**Abstract:**

To investigate the ecological impacts of macroalgae in the framework of shifting global CO_2_ concentrations, we conducted a study utilizing *Ulva fasciata* and *Sargassum horneri* specimens sourced from the Ma’an Archipelago in Zhejiang Province on how ocean acidification (OA) and temperature changes interact to affect the photosynthetic physiological responses of macroalgae. The results of the study showed that OA reduced the tolerance of *U. fasciata* to bright light at 20 °C, resulting in more pronounced photoinhibition, while 15 °C caused significant inhibition of *U. fasciata*, reducing its growth and photosynthetic activity, but OA alleviated the inhibition and promoted the growth of the alga to a certain extent. The tolerance of *S. horneri* to bright light was also reduced at 20 °C; the inhibition was relieved at 15 °C, and the OA further improved the algal growth. The Relative Growth Rate (*RGR*), photosynthetic pigment content, and the release of the dissolved organic carbon (DOC) of *U. fasciata* were mainly affected by the change in temperature; the growth of the alga and the synthesis of metabolites were more favored by 20 °C. A similar temperature dependence was observed for *S. horneri*, with faster growth and high metabolism at 15 °C. Our results suggest that OA reduces the tolerance of macroalgae to high light at suitable growth temperatures; however, at unsuitable growth temperatures, OA effectively mitigates this inhibitory effect and promotes algal growth.

## 1. Introduction

In recent years, with rapid economic development, the global environment has undergone swift transformations, particularly with the concerning escalation of CO_2_ levels in the atmosphere, posing a critical challenge to the sustainable progress of humanity. Gattuso anticipates a potential surge in atmospheric CO_2_ concentrations to 1000 μatm by the century’s conclusion [1]. Primarily absorbed by oceans, escalating atmospheric CO_2_ quantities will lead to a concurrent uptick in oceanic absorption, catalyzing a pH decline in surface oceanic waters—a phenomenon known as ocean acidification [2]. Moreover, the mounting atmospheric CO_2_ load will further intensify global warming, elevating the temperatures of oceanic surface waters [3,4]. Notably, within coastal ecosystems, intertidal macroalgae exhibit heightened sensitivity to fluctuating oceanic CO_2_ levels. This heightened sensitivity stems from the fact that macroalgae typically complete their entire life cycle within their habitats, thus rendering them particularly susceptible to enduring environmental vicissitudes in adjacent waters [5,6]. In addition, different species of macroalgae in the intertidal zone often show different responses to changes in CO_2_ concentration: no change [7], growth promotion [8], and growth inhibition [9]. The different responses are mainly related to macroalgae photosynthesis, as well as environmental adaptation [6].

Macroalgae thriving in coastal intertidal waters not only yield economic benefits within coastal seafood aquaculture but also serve as efficient absorbers of a myriad of pollutants abundant in elements such as N and P [10,11]. These macroalgae play a critical role in the marine ecosystem’s carbon cycle, effectively mitigating eutrophication while contributing positively to the regulation of marine ecological equilibrium and the moderation of atmospheric CO_2_ concentration increases. They stand as prime candidates for environmental rehabilitation in near-shore aquaculture marine regions [12]. Their growth and development primarily rely on the absorption and utilization of CO_2_ and HCO_3_^−^ from seawater to synthesize organic compounds. Ocean acidification can enhance the photosynthetic and growth rates of macroalgae to a certain extent [13]; however, it may diminish their photosynthetic efficiency when seawater pH falls below a specific threshold [14].

The response of macroalgae to temperature variations has been extensively scrutinized. Some research indicates that temperature impacts the growth, biochemical composition, and physiological processes of macroalgae [15,16]. Furthermore, changes in CO_2_ levels within seawater can influence how macroalgae adapt to external temperature shifts [17,18,19]. Investigations reveal that alterations in CO_2_ concentrations prompt biochemical changes in macroalgae, facilitating enhanced adaptation to temperature fluctuations in the external environment [19]. Numerous studies have delved into the photosynthetic physiology of macroalgae concerning temperature and CO_2_ alterations, examining facets like photosynthetic rates, pigments, and organic matter metabolism. However, these studies oftentimes isolate either factor individually, neglecting the combined effects of these influential variables. Gordillo proposed that the impact of CO_2_ concentrations becomes substantial when interacting with other factors [18]. Hence, it becomes imperative to explore the consequences of temperature and CO_2_ concentration fluctuations on macroalgae in a more integrated manner.

*Ulva fasciata* [11] and *Sargassum horneri* [20] stand out as the predominant macroalgae that naturally thrive in the coastal waters of southeastern China. These nutrient-rich species play pivotal roles across diverse sectors, including food, feed, medicine, and chemicals [17]. Possessing substantial biomass and thriving expansively, these key species not only serve crucial ecological functions as biological habitats but also prove pivotal in the restoration of macroalgae beds, a process of paramount significance for near-shore marine ecological initiatives [17,20]. Nestled in the northeastern region of Shengsi, Zhejiang Province, China, Ma’an Archipelago finds itself enveloped by a multitude of islands and abundant macroalgae. This investigation delves into the variations in photosynthetic activity exhibited by these two macroalgae species amidst the combined impacts of acidification and temperature shifts. Leveraging chlorophyll fluorescence techniques, we scrutinized changes in the biochemical compositions and physiological metabolism of *U. fasciata* and *S. horneri* in the waters surrounding Ma’an Archipelago, Zhejiang Province as the chosen test subjects. It is expected that insights into the effects of acidification and temperature synergism on the photosynthesis and physiological properties of *U. fasciata* and *S. horneri* will provide valuable data for further exploration in this area.

## 2. Materials and Methods

### 2.1. Sample Collection and Processing

The samples were collected from the intertidal waters of the Ma’an Archipelago, with *U. fasciata* originating from the coastal rocky reef zone and *S. horneri* from the mussel culture raft area. Subsequently, the collected macroalgae were cleansed in situ using seawater to eliminate any contaminants, such as floating debris or attached organisms. The indoor adaptive cultivation process lasted for 24 h. The acclimation was carried out using sterile seawater with salinity (28 ± 1‰) and temperatures (15 ± 1 °C) consistent with that of in situ seawater. The cultivation environment maintained a light intensity of 100 ± 5 μmol·m^−2^·s^−1^, determined through a field light intensity survey and the photosynthetic requirements of the macroalgae, alongside a photoperiod of L:D = 12 h:12 h.

### 2.2. Experimental Condition

After completing the acclimation phase, compliant samples (2 ± 0.005 g) were chosen from the collected macroalgae for subsequent experimentation. These selected samples were positioned in 2 L round-bottomed flasks, into which 1.2 L of sterile seawater was introduced along with an appropriate quantity of nutrient solution to avert any potential nutrient deficiencies (NH_4_: 150 mmol/L, NO_3_: 2100 mmol/L, P: 150 mmol/L, K: 900 mmol/L, Ca: 600 mmol/L, Mg: 300 mmol/L, and S: 300 mmol/L, adding about 2 mL). The flasks were then transferred to a CO_2_ incubator for the cultivation process. Acidification levels were precisely calibrated based on the present atmospheric CO_2_ concentration and Gattuso’s projections for CO_2_ levels by the century’s conclusion [1]: 400 μL·L^−1^ (blank treatment/LC) and 1000 μL·L^−1^ (acidification treatment/HC). Temperature settings were based on the seawater temperature at the time of sample collection (April and May) and the contribution of rising CO_2_ concentrations to global warming: 15 °C (natural temperature/LT) and 20 °C (warming temperature/HT). Each of the four cultivation conditions involved three sample groupings to study the combined effects of CO_2_ concentration and temperature, while a control group contained sterile seawater without any samples under each cultivation condition. Lighting conditions and photoperiod remained consistent with the acclimatization phase throughout the 7-day cultivation duration. Parameters of algae and seawater were measured during the early (day 1), middle (days 3 and 5), and later (day 7) of cultivation.

### 2.3. Rapid Light Curve and Fluorescence Induction Parameters

The Rapid Light Curve (*RLC*) of the samples was determined for each cultivation condition 1 h after the start of light exposure at a set number of cultivation days. A chlorophyll fluorometer (WALZ DIVING PAM, Effeltrich, Germany) was used to provide the samples sequentially with a total of 8 gradients of 0–2000 μmol·m^−2^·s^−1^ of photochemical light, with an interval of 20 s between two photochemical lights. After the determination, the RLC of each sample was derived from the obtained Relative Electron Transfer Rate (*rETR*) fitted by the exponential function formula, with reference to the following exponential function formula:

(1) $$rETR=rETRm(1-e^{-\alpha \cdot \mathrm{PAR}/rETRm}){\;e}^{-\beta \cdot \mathrm{PAR}/rETRm}$$

where PAR is the corresponding light intensity at the time of the measurement of the sample, *rETR*. Other relevant parameters included the maximum relative electron transfer rate (*rETRm*), macroalgae light energy utilization efficiency (α), photoinhibition parameter (β), and half-saturated light intensity (*E_k_*) were obtained with the *RLC*.

The samples were dark-adapted for 1–2 h after the end of light exposure, and the fluorescence induction parameters were determined using a chlorophyll fluorometer. The maximum quantum yield (*Fv*/*Fm*) of photosystem II (PSII) was calculated by the following equation:

*Fv/Fm = (Fm − Fo)/Fm* (2)

where *Fv* is the dark-adapted variable fluorescence value, *Fm* is the maximum fluorescence value at the dark-adapted saturating light intensity, and *Fo* is the dark-adapted initial fluorescence value. The effective quantum yield (*Fv*’/*Fm*’) of PSII was calculated by the following equation:

*Fv’/Fm’ = (Fm’ − Fo’)/Fm’* (3)

where *Fm*’ is the maximum chlorophyll fluorescence at a preset level of photochemical light, and *Fo*’ is the minimum chlorophyll fluorescence at a preset level of photochemical light. Photochemical quenching (*qP*) and non-photochemical quenching (*NPQ*) were calculated by the following equations:

*qP* = (*Fm*’ − *F*)/(*Fm*’ − *Fo*) (4)

*NPQ* = (*Fm* − *Fm*’)/*Fm*’ (5)

### 2.4. Relative Growth Rate

After the determination of the chlorophyll fluorescence parameters of the samples, the surface of the samples was dried with absorbent paper, the weight (fresh weight, Fw) was determined by using an electronic balance with 3 decimal places, and the Relative Growth Rate (RGR) of the macroalgae was calculated using the following formula:

*RGR* = [(*W_t_*/*W_o_*) − 1]/t (6)

where *W_o_* is the initial weight of the sample (Fw), and *W_t_* is the weight of the sample on day t (Fw).

### 2.5. Dissolved Organic Carbon Release

After 1 h of light initiation at the set number of cultivation days, the culturing seawater in each group of flasks was measured, and the DOC content of the seawater was determined. Seawater from 50 mL flasks of each group was aspirated and filtered using cauterized GF/F (Whatman, Maidstone, Kent, UK) glass fiber filtration membranes, after which, the resulting filtrate was subjected to DOC concentration determination with a Total Organic Carbon analyzer (Shimadzu TOC-L, Otsu, Japan). Each set of experiments was replicated 3 times.

### 2.6. Photosynthetic Pigment Content

After the growth parameters were determined, 0.2 ± 0.005 g samples (Fw) were accurately weighed by using an electronic balance with 3 decimal places, cut with scissors, and put into a mortar, adding appropriate amounts of quartz sand with 80% acetone, grinding thoroughly, followed by fixing them to 15 mL and then keeping them at 4 °C for 24 h in the dark; after that, the samples were centrifuged with a freezing centrifuge at 5000 r/min for 10 min at 4 °C. Then, the supernatant was taken, and the absorbance value was determined with a UV spectrophotometer (Mapada UV-3200, Shanghai, China). Each set of experiments was replicated 3 times. The chlorophyll a (Chl-a) and carotenoid (Car) contents were calculated according to Hellebustand Craigie [21]:

Chl-a (mg/g) = (11.85 × *A*_665_ − 1.54 × *A*_647_ − 0.08 × *A*_639_) × *V*/*W* (7)

Car (mg/g) = 7.6 × (*A*_480_ − 1.49 × *A*_510_) × *V*/*W* (8)

where *A* denotes the absorbance value of the supernatant at different wavelengths of light, *V* denotes the volume of the fixed volume, and *W* denotes the fresh weight of the macroalgae.

### 2.7. Statistical Analysis

The Excel 2019 software was used for experimental data processing, and Origin 2023 was used for plotting; one-way ANOVA analysis was performed using Tukey’s test, and two-way ANOVA analysis was performed to analyze the interaction of different CO_2_ concentrations and temperature changes in the macroalgae (Signifcance levels were set at *p* < 0.05). The results of the experimental measurements are expressed as the mean and standard deviation.

## 3. Results

### 3.1. Changes in Chlorophyll Fluorescence Parameters

**Figure 1:**
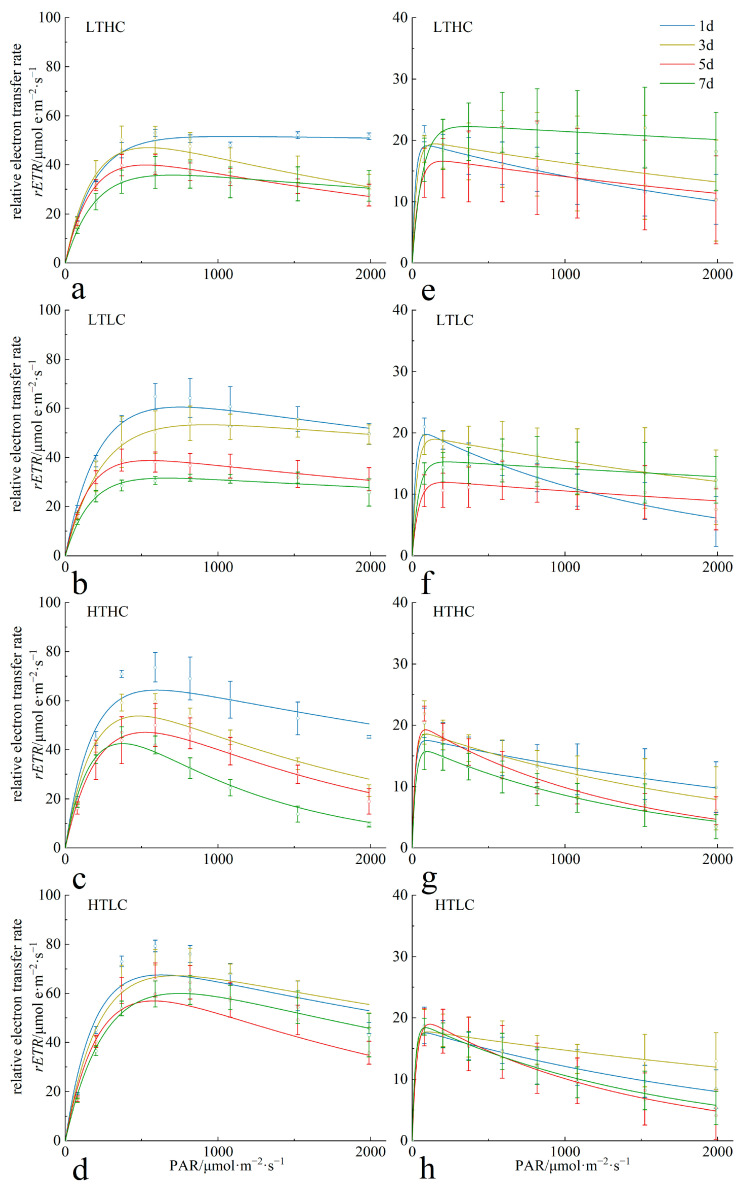
Changes in *RLC* of *U. fasciata* (**a**–**d**) and *S. horneri* (**e**–**h**) during cultivation in different conditions. Cultivation conditions for each group: LTLC (15 °C and 1000 μL·L^−1^), LTLC (15 °C and 400 μL·L^−1^), HTHC (20 °C and 1000 μL·L^−1^), and HTLC (20 °C and 400 μL·L^−1^). The error line is the SD.

The acidification effect induced by elevated CO_2_ concentration on the *RLC* of *U. fasciata* varied significantly at different temperatures. At 15 °C, the effect of the acidification treatment (1000 μL·L^−1^) was not significant during the cultivation period (*p* > 0.05), and relative to the blank treatment (400 μL·L^−1^), the *rETR* of the acidification treatment group showed a decrease in the early stage of cultivation, but in the middle and later stages of cultivation, the CO_2_ concentration did not have a significant effect on the *rETR* of *U. fasciata* (*p* < 0.05). The effect of the acidification treatment on *U. fasciata* was more obvious at 20 °C. The *rETR* of the algae decreased with the increase in cultivation time and was lower than that of the blank treatment group at the same cultivation time; moreover, the acidification treatment further aggravated the photoinhibitory effect of the algae, which led to a larger decrease in the *rETR* (Figure 1, left side).

At different temperatures, the acidification effect induced by the elevated CO_2_ concentration did not have a significant effect on the *RLC* of *S. horneri* (*p* < 0.05). At 15 °C, the *rETR* of *S. horneri* increased in both groups, but the increase was larger in the acidification treatment group. At 20 °C, elevated CO_2_ concentrations inhibited the growth of *S. horneri* and exacerbated the photoinhibition of the alga in low light (Figure 1, right side).

**Figure 2:**
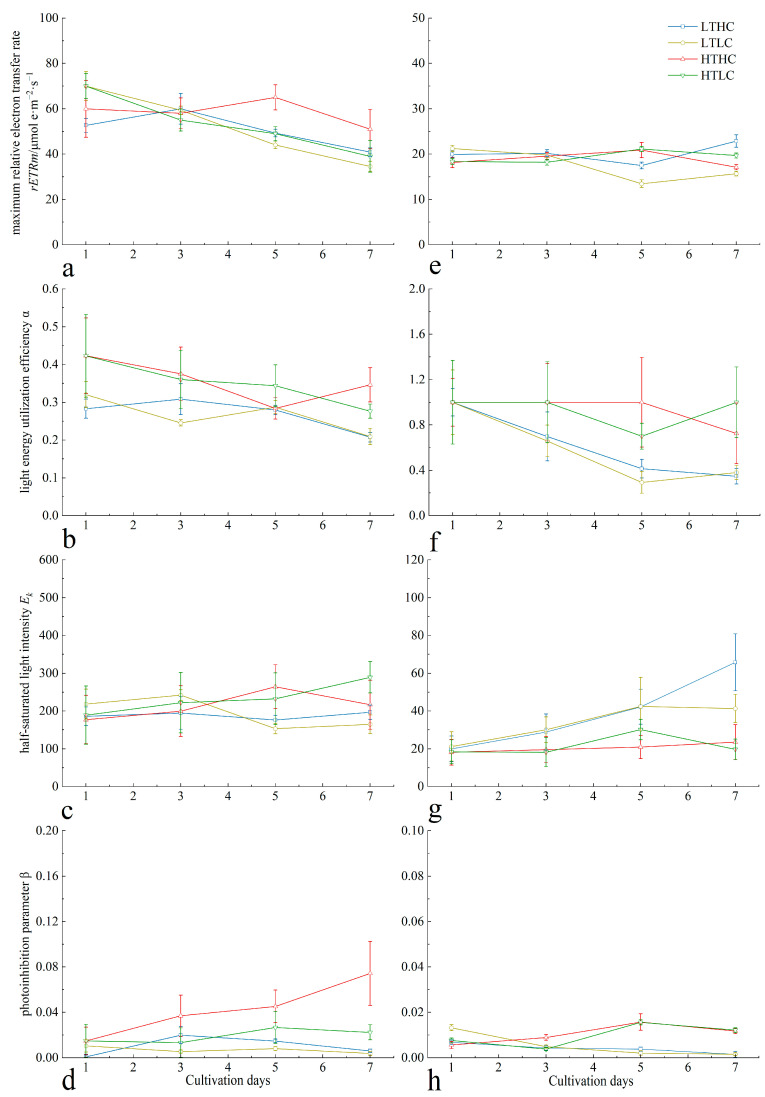
Changes in *RLC*-related parameters of *U. fasciata* (**a**–**d**) and *S. horneri* (**e**–**h**) during cultivation under different conditions. Cultivation conditions for each group: LTLC (15 °C and 1000 μL·L^−1^), LTLC (15 °C and 400 μL·L^−1^), HTHC (20 °C and 1000 μL·L^−1^), and HTLC (20 °C and 400 μL·L^−1^). The error line is the SD.

Acidification treatments generally suppressed the maximum relative electron transfer rate (*rETRm*) of *U. fasciata* at different temperatures in the early stage of cultivation, but in the later stage of cultivation, the acidification treatments presented a promotional effect on the *rETRm* of *U. fasciata* at 20 °C; on the other hand, the *rETRms* of the blank treatment group at different temperatures all presented a continuous decreasing trend (Figure 2a). Relative to changes in CO_2_ concentrations, the light energy utilization efficiency (α) and half-saturated light intensity (*E_k_*) of *U. fasciata* were mainly affected by changes in temperature (Figure 2b). *U. fasciata* cultured at different CO_2_ concentrations at 20 °C had higher α than those cultured at 15 °C during the cultivation period. The acidification treatment had no significant effect on the *E_k_* of *U. fasciata* at different temperatures (*p* > 0.05). The *E_k_* was higher at 15 °C for the early stage of cultivation and at 20 °C for the later stage of cultivation (Figure 2c). Temperature changes had an effect on the trend of the photoinhibition parameter (β) of *U. fasciata*. At 20 °C, the acidification treatment resulted in a sustained increase in β with cultivation time, which was higher than that of the blank treatment group at the same cultivation time; at 15 °C, the CO_2_ concentration had no significant effect on β (Figure 2d).

Likewise, the variation trend of *rETRm* in *S. horneri* closely paralleled its *RLC*. In the early stage of cultivation, *rETRm* was not affected by changes in temperature or CO_2_ concentration, but in the middle stage of cultivation, the *rETRm* of *S. horneri* at 15 °C decreased and then finally returned to normal levels in the later stage of cultivation (Figure 2e). The α, *E_k_*, and β of *S. horneri*, on the other hand, were mainly affected by temperature changes during the cultivation period (*p* < 0.05), and the effect of CO_2_ concentration changes on them was not significant. At 15 °C, the α of *S. horneri* cultured with different CO_2_ concentrations showed a significant decreasing trend in the middle and later stages of cultivation, while the α at 20 °C was more stable (Figure 2f). The *E_k_* showed a continuous increase at 15 °C, which was higher than that of *S. horneri* at 20 °C during the same cultivation time throughout the cultivation period, but the *E_k_* was more stable at 20 °C (Figure 2g). The acidification treatment had no significant effect on the β of *S. horneri* (*p* > 0.05). The β of the algae was higher at 15 °C in the early stage of cultivation and at 20 °C in the later stage of cultivation (Figure 2h).

**Figure 3:**
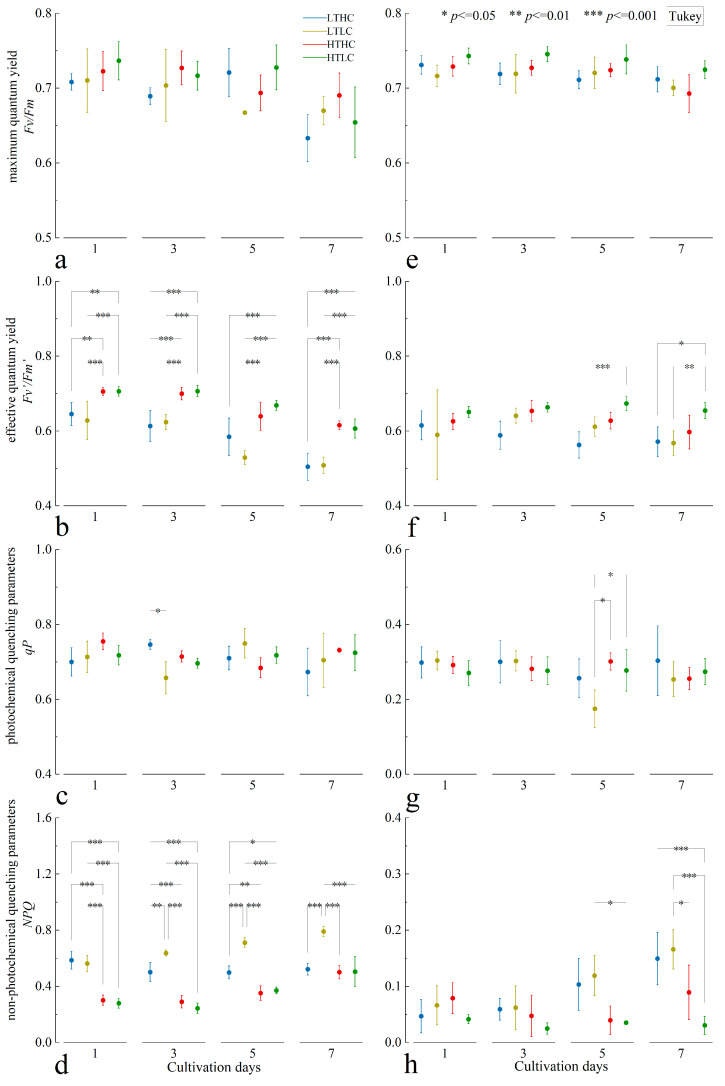
Changes in fluorescence induction parameters of *U. fasciata* (**a**–**d**) and *S. horneri* (**e**–**h**). Cultivation conditions for each group: LTLC (15 °C and 1000 μL·L^−1^), LTLC (15 °C and 400 μL·L^−1^), HTHC (20 °C and 1000 μL·L^−1^), and HTLC (20 °C and 400 μL·L^−1^). The error line is the SD.

The results show that the maximum quantum yield (*Fm*/*Fv*) of *U. fasciata* was more stable in the early and middle stages of cultivation and showed a decreasing trend in the later stage of cultivation under different cultivation conditions, but the statistical analyses showed that the temperature and the CO_2_ concentration did not significantly affect the *Fm*/*Fv* of *U. fasciata* during the same cultivation time (*p* > 0.05) (Figure 3a). The effective quantum yield (*Fv’*/*Fm’*) of *U. fasciata* showed a decreasing trend under different cultivation conditions, and the *Fv’*/*Fm’* was mainly affected by the temperature throughout the whole cultivation period; on the other hand, the change in the CO_2_ concentration did not have a significant effect on it. The *Fv’*/*Fm’* at 20 °C was higher than that of the *U. fasciata* at 15 °C during the same cultivation time (Figure 3b). The trend of the photochemical quenching parameter (*qP*) of *U. fasciata* was similar to that of *Fm*/*Fv*, and *qP* was more stable throughout the cultivation period under different cultivation conditions, with less of an effect from temperature and CO_2_ concentration on *qP* (Figure 3c). In addition, the non-photochemical quenching parameter (*NPQ*) of the blank treatment group showed a continuous increase throughout the cultivation period at 15 °C, whereas the changes in *NPQ* were not significant under other cultivation conditions. Temperature changes significantly affected the *NPQ* of *U. fasciata* at different CO_2_ concentrations (*p* < 0.05), but the acidification treatment only had a more significant effect on *NPQ* at 15 °C (Figure 3d).

Under different cultivation conditions, the *Fm*/*Fv* of *S. horneri* was relatively stable throughout the cultivation period, and the effects of temperature and CO_2_ concentration on *Fm*/*Fv* were not significant (Figure 3e). The *Fv’*/*Fm’* of *S. horneri* was relatively stable in the early and middle stages of cultivation and was not affected by the cultivation conditions. However, as the cultivation progressed, the *Fv’*/*Fm’* of the blank treatment group at 20 °C showed a significant difference from that of *S. horneri* at 15 °C (*p* < 0.05), and throughout the whole cultivation period, the *Fv’*/*Fm’* of *S. horneri* at 20 °C was higher than that of *S. horneri* at 15 °C in the same cultivation time (Figure 3f). The trend in *qP* changes in *S. horneri* was similar to that of *U. fasciata*, with *qP* being more stable throughout the cultivation period under different cultivation conditions, while the effects of temperature and CO_2_ concentration on *qP* were smaller (Figure 3g). The *NPQ* of *S. horneri* at 15 °C showed a high level and a continuous increase with the cultivation time, while the *NPQ* at 20 °C was lower and did not change significantly, showing some differences compared to 15 °C in the middle and later stages of cultivation (Figure 3h).

### 3.2. Changes in RGR and DOC Release

**Figure 4:**
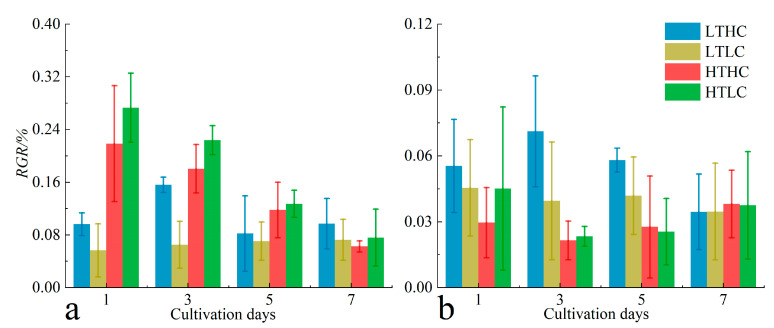
Changes in Relative Growth Rate (*RGR*) of *U. fasciata* (**a**) and *S. horneri* (**b**) during cultivation under different conditions. Cultivation conditions for each group: LTLC (15 °C and 1000 μL·L^−1^), LTLC (15 °C and 400 μL·L^−1^), HTHC (20 °C and 1000 μL·L^−1^), and HTLC (20 °C and 400 μL·L^−1^). The error line is the SD.

There were some differences in the effect of the acidification treatments on the *RGR* of *U. fasciata* at different cultivation temperatures. At 20 °C, the acidification treatment inhibited the growth of *U. fasciata*. At 15 °C, the acidification treatment promoted growth. In addition, the *RGR* at 20 °C was higher than that at 15 °C during the same cultivation time in both the early and middle stages of cultivation but was slightly lower than that at 15 °C in the later stages of cultivation (Figure 4a). At the end of cultivation, the interaction of different cultivation conditions did not significantly affect the *RGR* of *U. fasciata* (*p* > 0.05). The effect of the acidification treatments on the *RGR* of *S. horneri* was more similar to that of *U. fasciata*. Acidification treatments at 15 °C promoted growth in the early and middle stages of cultivation. The blank treatment group at 20 °C had a higher *RGR* in the early stage of cultivation, but the *RGR* of *S. horneri* at different CO_2_ concentrations converged as the cultivation time progressed. In addition, the *RGR* of *S. horneri* at 15 °C was higher than that at 20 °C during the same cultivation time in both the early and middle stages of cultivation, but in the later stage of cultivation, the *RGR* under each cultivation condition was more similar and did not show a significant difference (*p* > 0.05) (Figure 4b).

**Figure 5:**
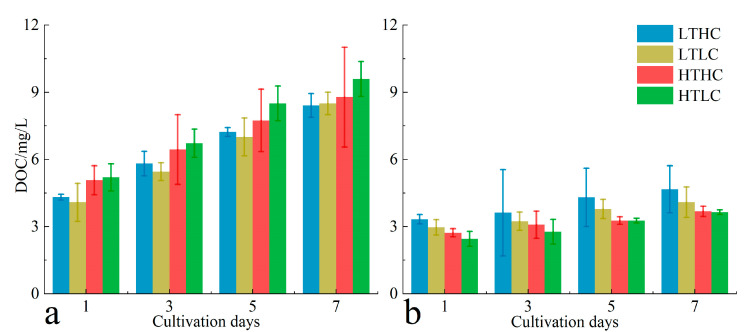
Changes in the release of dissolved organic carbon (DOC) from seawater during cultivation of *U. fasciata* (**a**) and *S. horneri* (**b**) under different conditions. Cultivation conditions for each group: LTLC (15 °C and 1000 μL·L^−1^), LTLC (15 °C and 400 μL·L^−1^), HTHC (20 °C and 1000 μL·L^−1^), and HTLC (20 °C and 400 μL·L^−1^). The error line is the SD.

The DOC release of *U. fasciata* showed a similar trend to its *RGR*. At 20 °C, the DOC release of the acidified treatment group was lower than that of the blank treatment group during the same cultivation time throughout the cultivation period. At 15 °C, the acidification treatment promoted the release of DOC from *U. fasciata* to external seawater in the early and middle stages of cultivation, but the DOC release from *U. fasciata* treated with different CO_2_ concentrations was more similar in the later stages of cultivation as the cultivation time progressed. At the end of cultivation, the DOC release from *U. fasciata* was significantly affected by the different cultivation conditions, as well as by the interaction (*p* > 0.05). In addition, the DOC release at 20 °C was higher than that of *U. fasciata* at 15 °C during the same cultivation time (Figure 5a). At 15 °C, the DOC release of *S. horneri* was higher in the acidified treatment group than in the blank treatment group during the same cultivation time throughout the cultivation period. At 20 °C, the DOC release of the acidified treatment group was higher than that of the blank treatment group at the same cultivation time in the early and middle stages of cultivation, but as the cultivation time progressed, the DOC release amounts of the two groups of *S. horneri* become closer in the later stages of cultivation. Changes in CO_2_ concentration and temperature did not significantly affect the DOC release from *S. horneri* at the end of the cultivation (*p* > 0.05). In addition, the DOC release was higher at 15 °C than at 20 °C for the same cultivation time for *S. horneri* (Figure 5b).

### 3.3. Changes in Photosynthetic Pigment Content

**Figure 6:**
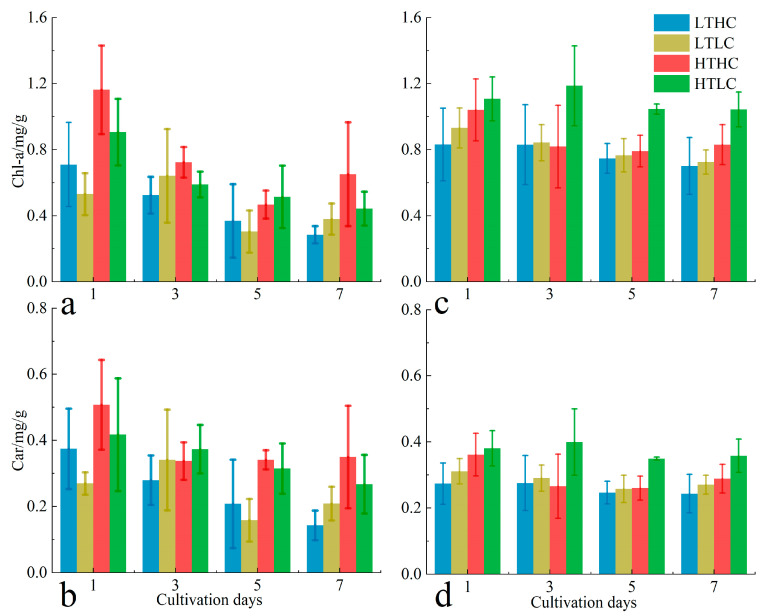
Changes in chlorophyll a (Chl-a) and carotenoids (Car) of *U. fasciata* ((**a**) Chl-a; (**b**) Car) and *S. horneri* ((**c**) Chl-a; (**d**) Car) during cultivation in different conditions. Cultivation conditions for each group: LTLC (15 °C and 1000 μL·L^−1^), LTLC (15 °C and 400 μL·L^−1^), HTHC (20 °C and 1000 μL·L^−1^), and HTLC (20 °C and 400 μL·L^−1^). The error line is the SD.

Under different cultivation conditions, the Chl-a content of *U. fasciata* mostly showed a decreasing trend. At 20 °C, the Chl-a content of the acidification treatment group was mostly higher than that of the blank treatment group during the whole cultivation period; the Chl-a content in the acidification treatment group showed a decreasing and then increasing trend, but the blank treatment group showed a continuous decreasing trend throughout the cultivation period. At 15 °C, the CO_2_ concentration did not cause significant differences in the Chl-a content of *U. fasciata*; the acidification treatment group showed a continuous decreasing trend in Chl-a content, while the blank treatment group did not show any significant changing trend. In addition, the Chl-a content at 20 °C was mostly higher than that of *U. fasciata* at 15 °C during the same cultivation time (Figure 6a). The trend in the Car content of *U. fasciata* under each cultivation condition was more similar to that of its Chl-a content, which was mainly affected by temperature changes throughout the cultivation period, and the Car content at 20 °C was mostly higher than that of *U. fasciata* at 15 °C during the same cultivation time (Figure 6b).

The trend in the photosynthetic pigment content of *S. horneri* differed somewhat from that of *U. fasciata*. At 20 °C, the Chl-a content of the acidification treatment group was lower than that of the blank treatment group in the same cultivation time throughout the cultivation period; the Chl-a content of the acidification treatment group showed a tendency to increase and then decrease, whereas the Chl-a content of the blank treatment group showed a tendency to slowly decrease. At 15 °C, the acidification treatment caused the Chl-a content of *S. horneri* to be lower than that of the blank treatment group in the early stage of cultivation, but the effect of CO_2_ concentration on the Chl-a content gradually decreased with the cultivation time; the Chl-a content of *S. horneri* incubated with different CO_2_ concentrations showed a slow decreasing tendency throughout the whole cultivation period. In addition, the Chl-a content at 20 °C was mostly higher than that of *S. horneri* at 15 °C during the same cultivation time (Figure 6c). The trend in the Car content of *S. horneri* was similar to that of its Chl-a content under all cultivation conditions. The highest levels of Chl-a and Car were found in the blank treatment group at 20 °C and were at high levels throughout the cultivation period (Figure 6d). As with *U. fasciata*, the interaction between CO_2_ concentration and temperature changes did not significantly affect the photosynthetic pigment content of the two macroalgae at the end of the cultivation (*p* > 0.05).

## 4. Discussion

### 4.1. Changes in Fluorescence Parameters and Growth of Macroalgae

The continued rise in atmospheric CO_2_ concentrations has led to ocean acidification and global warming, which, in turn, affects the lives of most marine organisms. Among them, organisms in offshore intertidal waters show stronger environmental adaptability than those in pelagic waters [22], and intertidal macroalgae are often used as research models when exploring the effects of environmental changes on intertidal marine organisms [23]. The results of this study showed that the *Fv*/*Fm* and *Fv’*/*Fm’* of *U. fasciata* at 15 °C were lower than those at 20 °C during the same cultivation time throughout an entire cultivation period, suggesting that *U. fasciata* was subjected to the stressful effects of low temperature. In addition, the rise in *NPQ* and the decrease in *rETRm* in the blank treatment group at 15 °C suggests that *U. fasciata* protects its PSII structure by increasing the heat released from non-photochemical processes as a means of depleting captured light energy, allowing it to maintain a high photosynthetic capacity and tolerance [24]. However, under an acidification treatment at 15 °C, the *NPQ* of *U. fasciata* was more stable throughout the cultivation period, and the *RGR* was also higher than that of the blank treatment group, suggesting that the acidification treatment could alleviate the stressful effects of low temperature. This may be due to the fact that the acidification treatment increased the amount of CO_2_ in the cultured seawater [25]. Under high CO_2_ concentrations, *U. fasciata* regulates the energy required for CO_2_ concentration mechanisms (CCMs) [4] to better cope with the stressful effects of low temperatures. Higher cultivation temperatures promoted the growth and photosynthesis of *U. fasciata* relative to 15 °C. It has been shown that the suitable temperature for the growth of *U. fasciata* under natural conditions is around 25 °C [20]. This experiment also found that 20 °C was more favorable for the growth of *U. fasciata*, and the fluorescence parameters were at a higher level, which Fu et al. attributed to the higher activity of the key enzymes of photosynthesis in macroalgae at higher temperatures [26]. However, at this temperature, the acidification treatment did not significantly affect the growth and photosynthetic use efficiency of *U. fasciata*. In the present study, it was found that the *RGR* and the fluorescence parameters of the acidified treatment group at 20 °C did not show any significant difference from the blank treatment group. According to Wu et al. [6], the supply of CO_2_ promotes the growth and photosynthesis of macroalgae at suitable temperatures, but their photosystems also carry out a coordinated action to integrate the carbon assimilation process affected by temperature. Furthermore, it has also been shown that an appropriate increase in seawater CO_2_ concentrations does not significantly affect macroalgae grown under saturated light conditions [26]. Excessive CO_2_ concentrations at 20 °C increased the *rETRm* and β of *U. fasciata* during long-term cultivation, also indicating that acidification treatments increase the photosynthetic potential of *U. fasciata* but decrease the tolerance of algae to high light.

At 15 °C, the *RGR* of *S. horneri* was mostly higher than that of *S. horneri* at 20 °C in the early stage of cultivation, but the *RGR* of *S. horneri* at 15 °C decreased rapidly with cultivation time in the later stage of cultivation. Therefore, subsequent studies found that *S. horneri* increased its demand for light energy in order to maintain normal growth, and the *E_k_* and *NPQ* of *S. horneri* at 15 °C gradually increased with cultivation time, indicating that *S. horneri* enhanced its protective mechanism to prevent excessive light energy from destroying the structure of PSII while improving its ability to capture light energy [13]. In addition, the effect of the acidification treatment on the fluorescence parameters of *S. horneri* was not significant at 15 °C, but the *RGR* was significantly higher than that of the blank treatment group in the early and middle stages of cultivation. This may be because the CO_2_ concentration in the seawater of the blank treatment was low, which failed to meet the demands of photosynthesis for *S. horneri*, while the acidification treatment allowed *S. horneri* to obtain more CO_2_, which, in turn, promoted the growth of *S. horneri* [27], but with the time of cultivation, excess CO_2_ produced a negative feedback regulation on *S. horneri* and inhibited its growth. [28]. It has been pointed out that 11–16 °C is the optimal range for *S. horneri* growth and reproduction [29,30]. The results of the present study showed that 20 °C did not significantly affect the levels of the fluorescence parameters *rETRm*, *Fv*/*Fm*, and *Fv’*/*Fm’* in *S. horneri*, suggesting that higher temperatures do not diminish the photosynthetic activity of *S. horneri*. However, the β of *S. horneri* continued to rise at higher temperatures with cultivation time, suggesting that 20 °C may have attenuated light energy capture and light acclimation in *S. horneri*. In addition, at 20 °C, the acidification treatment increased the *NPQ* of *S. horneri*, causing it to release excess energy in the form of heat dissipation, alleviating the stressful effects of high temperature to some extent [6].

### 4.2. Changes in Biochemical Characterization Parameters of Macroalgae

The 15 °C cultivation temperature resulted in lower DOC release from *U. fasciata*, possibly due to lower temperatures inhibiting the activity of key enzymes in photosynthesis [24]. The acidification treatment had a mitigating effect on the stressful effects of low temperature, resulting in a higher level of DOC release in the acidification treatment group at 15 °C. In addition, the macroalgae adapted to the low-temperature environment over the cultivation time, resulting in a convergence of DOC release from *U. fasciata* at different CO_2_ concentrations. Rogers also found that too much CO_2_ inhibits the activity of Rubisco enzymes at suitable temperatures, which, in turn, inhibits the rate of assimilation of inorganic carbon by macroalgae [31]. Therefore, the activity of *U. fasciata* photosynthesis enzymes is saturated under acidification treatments at 20 °C, and an excessive CO_2_ concentration decreases the rate of synthesis of metabolites such as proteins [32] and lipids [33]. The DOC release from *S. horneri* was at a higher level at 15 °C, which was attributed by Marañón [34] to the higher *RGR* of *S. horneri* at 15 °C. The acidification treatment increased the CO_2_ concentration in the cultured seawater, which promoted the growth of *S. horneri* and also increased the synthesis rate of its own metabolites, and the DOC release from *S. horneri* reached its highest level under the acidification treatment at 15 °C. In addition, different CO_2_ concentrations did not produce a significant effect on the DOC release under 20 °C cultivation conditions, probably because under the stress of high temperature *S. horneri* allocated more energy to the synthesis of photosynthetic pigments, and thus, the acidification treatment did not significantly increase the activity of metabolic enzymes in the cells [35].

The photosynthetic pigment content reflects changes in the ability of macroalgae to regulate their own physiological responses [36]. It has been shown that macroalgae are able to self-regulate the content of pigments to adapt to changes in the external growth environment [37]. In this study, we showed that the photosynthetic pigment content of *U. fasciata* at 15 °C was lower than that of *U. fasciata* at 20 °C during the same cultivation period. This may be due to *U. fasciata* cells at 15 °C reducing the synthesis of photosynthetic pigments to decrease the absorption area of Photosystem I (PSI) and the activity ratio of PSI to PSII. This reduction leads to a decrease in the synthesis of high-energy compound adenosine triphosphate (ATP) to conserve energy for maintaining normal life activities [27]. Under acidification treatment conditions at 15 °C, low temperatures inhibit the photosynthesis of *U. fasciata*, so even though the amount of dissolved inorganic carbon (DIC) available in cultured seawater is increased, the photosynthetic capacity of *U. fasciata* is essentially saturated so that it regulates its own biochemical reactions without significantly altering photosynthetic pigment content [38]. However, a cultivation temperature of 20 °C promoted the activity of enzymes, as well as other physiological components, in *U. fasciata*, resulting in the need to synthesize more Chl-a and Car in the 20 °C acidification treatment group in order to maintain a higher photosynthetic rate for more light energy. *S. horneri* is a light-adapted macroalga that grows well under higher seawater transparency and shallow cultivation conditions; thus, its growth is significantly affected under lower seawater transparency and unfavorable light conditions, which may even lead to slow growth or death [29]. Where cultivation light is not the maximum light intensity required for *S. horneri*, the stressful effects of higher cultivation temperatures can force *S. horneri* to enhance its own light energy harvesting capacity by increasing the synthesis of photosynthetic pigments [39]. The present study also showed that the acidification treatment provided some relief from the effects of high-temperature stress on *S. horneri*. In addition, the high concentration of CO_2_ saves the energy required for *S. horneri*’s CCMs [40], allowing it to maintain normal levels of vital activity without synthesizing excessive photosynthetic pigments under low-temperature cultivation conditions [41].

## 5. Conclusions

At 20 °C, the growth metabolism of *U. fasciata* is at a relatively high level. The acidification treatment further enhances the photosynthesis of *U. fasciata* but at the same time weakens its tolerance to high light intensity. In contrast to *U. fasciata*, *S. horneri* is more adapted to low-temperature environments. At 15 °C, both the growth and adaptability of *S. horneri* are improved. Although the acidification treatment promotes the growth metabolism of *S. horneri*, it suppresses the synthesis of its photosynthetic pigments. Long-term cultivation results show that compared with *U. fasciata*, *S. horneri* exhibits greater adaptability to environmental changes. With the passage of cultivation time, the regulation of growth metabolism and energy required for synthesizing substances in *S. horneri* becomes more rational. This enables *S. horneri* to grow and develop without significant inhibition under different stress conditions, with no significant differences in its physiological characteristic parameters.

## Data Availability

The original contributions presented in this study are included in the article, and further inquiries can be directed to the corresponding author.
